# Establishment of monocular-limited photoreceptor degeneration models in rabbits

**DOI:** 10.1186/1471-2415-13-19

**Published:** 2013-05-17

**Authors:** Hitomi Isago, Eriko Sugano, Namie Murayama, Makoto Tamai, Hiroshi Tomita

**Affiliations:** 1 Laboratory of Visual Neuroscience, Department of Chemistry and Bioengineering, Iwate University Graduate School of Engineering, 4-3-5 Ueda Morioka, Iwate, 020-8551, Japan; 2 Tohoku University Graduate School of Medicine, 1-1 Seiryo Aoba Sendai Miyagi, Tohoku, 980-8574, Japan; 3 Clinical Research, Innovation and Education Center, Tohoku University Hospital, 1-1 Seiryo Aoba Sendai Miyagi, Tohoku, 980-8574, Japan

**Keywords:** Photoreceptor degeneration, Monocular, Pigmented rabbits, Verteporfin, Nitric oxide

## Abstract

**Background:**

Numerous rodent models of photoreceptor degeneration have been developed for the study of visual function. However, no viable model has been established in a species that is more closely related to *Homo sapiens*. Here, we present a rabbit model of monocular photoreceptor degeneration.

**Methods:**

We tested 2 chemicals, verteporfin and sodium nitroprusside (SNP), for developing a 1-eye limited photoreceptor degeneration model in pigmented rabbits. After the intravenous injection of verteporfin, the retina was exposed to light from a halogen lamp for 0, 10, 30, or 60 min. Alternately, 100 μL of various concentrations of sodium nitroprusside (0.1 mM, 0.5 mM, and 1 mM) were intravitreously injected into the rabbit eye. Retinal degeneration was evaluated by fundus photography, electroretinogram (ERG), and histological examinations.

**Results:**

Fundus photographs of animals in the verteporfin- or SNP-treated groups showed evidence of retinal degeneration. The severity of this degradation depended on the duration of light exposure and the concentration of SNP administered. The degeneration was clearly limited to the light-exposed areas in the verteporfin-treated groups. Extensive retinal atrophy was observed in the SNP-treated groups. The a- and b-wave amplitudes were dramatically decreased on the ERGs from SNP-treated groups. Histological examination revealed that either verteporfin or SNP induced severe photoreceptor degeneration. High-dose SNP treatment (1 mM) was also associated with inner retinal layer degeneration.

**Conclusions:**

Both SNP and verteporfin clearly caused photoreceptor degeneration without any effect on the contralateral eye. These compounds therefore represent valuable tools for the empirical investigation of visual function recovery. The findings will inform guidelines for clinical applications such as retinal prostheses, cell-based therapy, and gene therapy.

## Background

Retinitis pigmentosa (RP) is a degenerative disease of the retina that causes night blindness and ultimately a loss of peripheral and central vision [1]. Several genes responsible for RP have been identified, most of which are related to phototransduction pathways. However, these findings have not yet led to the discovery of effective treatments or prevention strategies. Retinal prostheses that elicit phosphenes by stimulating the remaining retinal neurons have been studied as potential tools to restore vision in these patients. Recently, clinical trials performed in the U.S. [2], Germany [3], and Japan [4] have reported successful results. Sasai et al. successfully created a complete, three-dimensional retina in vitro using embryonic stem cells [5]. Our group as well as investigations by Bi et al. have used gene therapy to restore vision [6-10]. Numerous similar efforts represent additional potential tools on the horizon of vision recovery.

Various rodent models of photoreceptor degeneration have been developed for the study of visual function. The Royal College of Surgeons (RCS) rats [11] carry a mutation in the Mertk gene that induces spontaneous photoreceptor degeneration [12]. Continuous light exposure is another technique used to investigate the mechanisms of photoreceptor degeneration. S334ter [13], P23H [14,15], rd, and rds [16,17] transgenic rats harbour mutations associated with human RP. Thus far, however, no model has been established in higher animals. The rabbit model of photoreceptor degeneration presented here would allow for experiments on complicated behavioural tasks that more closely simulate visual function in humans.

Here, we report 2 methods for generating a rabbit model of monocular photoreceptor degeneration. Verteporfin (Novartis AG, Bülach, Switzerland) is a photosensitising dye that is used clinically, without any adverse effects, for patients with subfoveal choroidal neovasucularisation (CNV). However it has been reported in pre-clinical studies that overdoses of verteporfin and/or extended light exposure induce photoreceptor and RPE cell damage [18]. Here, verteporfin is used for the purpose of the induction of local photoreceptor degeneration. Sodium nitroprusside (SNP) is used for the treatment of hypertension [19]. SNP breaks down in the blood and releases nitric oxide (NO), which works as a vasodilator. In the retina, excess NO induces photoreceptor degeneration. The intravitreous injection of SNP induces extensive photoreceptor degeneration.

Both verteporfin and SNP induce marked photoreceptor degeneration without any effect on the contralateral eye. The benefits of a monocular model of photoreceptor degeneration include use of the fellow eye as a control, which reduces the number of animals to be utilised for the experiment. Finally, the relatively large size of a rabbit vs. rodent eye renders surgery less challenging.

## Methods

Verteporfin was applied according to the manufacturer’s protocol, except for slight modifications. In our study, we used a halogen reflector lamp as the light source. In brief, a vial containing 2 mg of verteporfin was dissolved in 7 mL of sterile distilled water, then diluted with a 5% Otsuka glucose solution (Otsuka Pharmaceutical Co., Ltd, Tokyo) to adjust the concentration to 0.1 mg/mL. The solution (0.5 mg/kg) was injected intravenously at a speed of 1 mL/min. SNP was dissolved in saline, and various concentrations of SNP were injected intravitreously into the pigmented rabbit eyes.

Eighteen, male Dutch rabbits (kbl: Dutch, 1.5–2.0 kg) were used for experiments. These animals were used in accordance with the ARVO Statement for the Use of Animals in Ophthalmic and Vision Research and the Guidelines for Animal Experiments of Tohoku University. All animal experiments were conducted with the approval of the Animal Research Committee, Graduate School of Medicine, Tohoku University.

The rabbits were anesthetised by the intramuscular injection of a mixture of ketamine (66 mg/mL) and xylazine (33 mg/kg). To induce retinal degeneration using verteporfin, the retina was exposed to light from a halogen reflector lamp located 10 mm from the cornea (40000 lux; KL 2500 LCD, Carl Zeiss, Göttingen) after dilating the pupils with 1% atropine and 2.5% phenylephrine hydrochloride (Mydrin P; Santen Pharmaceutical Co., Ltd., Osaka, Japan). For the second model, after oxybuprocaine hydrochloride (0.4% Benoxil ophthalmic solution; Santen Pharmaceutical Co., Ltd., Osaka, Japan) was applied topically to the eye, using an operating microscope, a small incision was made in the conjunctiva to expose the sclera, and 100 μl of SNP solution was injected intravitreally through the ora serrata with a 30-gauge needle. Two weeks later, fundus photographs were obtained using a handy fundus camera (Genesis Df; Kowa, Tokyo, Japan). ERG recordings were obtained 1 month after the treatment.

ERGs were recorded using a Neuropack (MEB-9102; Nihon Kohden, Tokyo, Japan) as previously described [10]. Briefly, rabbits were dark-adapted overnight, pupils were dilated with 1% atropine and 2.5% phenylephrine hydrochloride, and the corneas were anaesthetised with 0.5% proparacaine hydrochloride. Small contact lenses with gold wire loops were placed on both corneas, and a silver wire reference electrode was placed subcutaneously between the eyes. Flash light stimuli with a duration of 10 ms were generated by pulse activation of a white LED. Full-field scotopic ERGs were recorded, band-pass filtered at 0.3–500 Hz, and averaged for 5 responses at each light intensity. The ground electrode clip was placed on the tail. Photic stimuli were generated by pulse activation of a white light-emitting diode (LED). A white LED (7500 Kelvin) was used for white stimuli; further, because a white colour LED includes multiple wavelengths, Kelvins were generally used as the unit of measurement.

The analysis of retinal morphology in verteporfin- and SNP-treated eyes was performed as previously described by Tomita et al. [9]. In brief, rabbits were sacrificed by the intravenous injection of pentobarbital. The eyes were enucleated, fixed, and embedded in paraffin. Three micrometre-thick sections of the retina were cut along the horizontal meridian and stained with haematoxylin and eosin.

Statistical analysis was performed using GraphPad Prism software (GraphPad Software, San Diego, CA). The criterion for statistical significance was *p* < 0.05. The statistical analysis was performed using an unpaired *t*-test.

## Results

**Figure 1:**
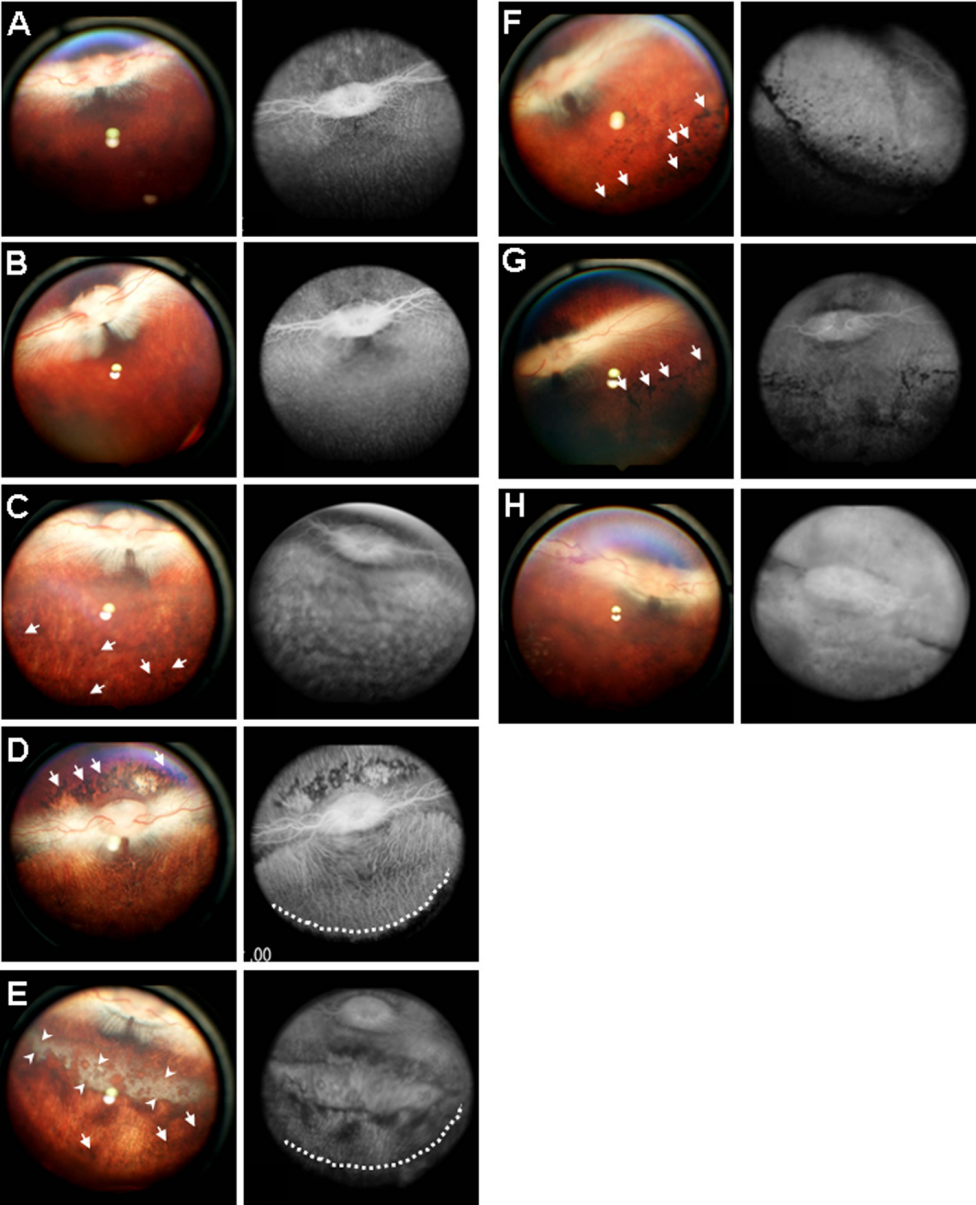
**Fundus photographs taken 2 weeks after treatment with PDT or intravitreal SNP.** The control fundus is shown in **A**. PDT was performed with 0.5 mg/kg verteporfin and a halogen reflector lamp for 0 (**B**), 10 (**C**), 30 (**D**), or 60 (**E**) min. One hundred microliters of SNP (**F**: 0.1 mM, **G**: 0.5 mM, **H**: 1 mM) was injected intravitreously into a rabbit eye. Arrows and arrowheads indicate the pigmentation and the area of severe degeneration, respectively. Lesions are clearly demarcated by a broken line.

There were no obvious changes between untreated- (Figure 1A) and verteporfin-treated retinas without light exposure (Figure 1B). Light exposure following verteporfin treatment (Figure 1C-E) or the intravitreous injection of SNP (Figure 1F-H) induced retinal atrophy. The degree of atrophy depended on the duration of light exposure or SNP concentration. When the verteporfin-treated retina was exposed to light, for even as little as 10 min, pigmentation of the retinal pigment epithelial (RPE) cells was observed clearly (Figure 1C). As the duration of light exposure was extended, the pigmentation became more obvious and was labelled as RPE atrophy (Figure 1C-E). The lesions induced by verteporfin with light exposure were clearly restricted to the area exposed to light (Figure 1D). However, the degeneration induced by SNP extended to the peripheral retina (Figure 1F-H). Fluorescence angiography showed some haemorrhage around the optic nerve in eyes injected with 1 mM of SNP (Figure 1H).

**Figure 2:**
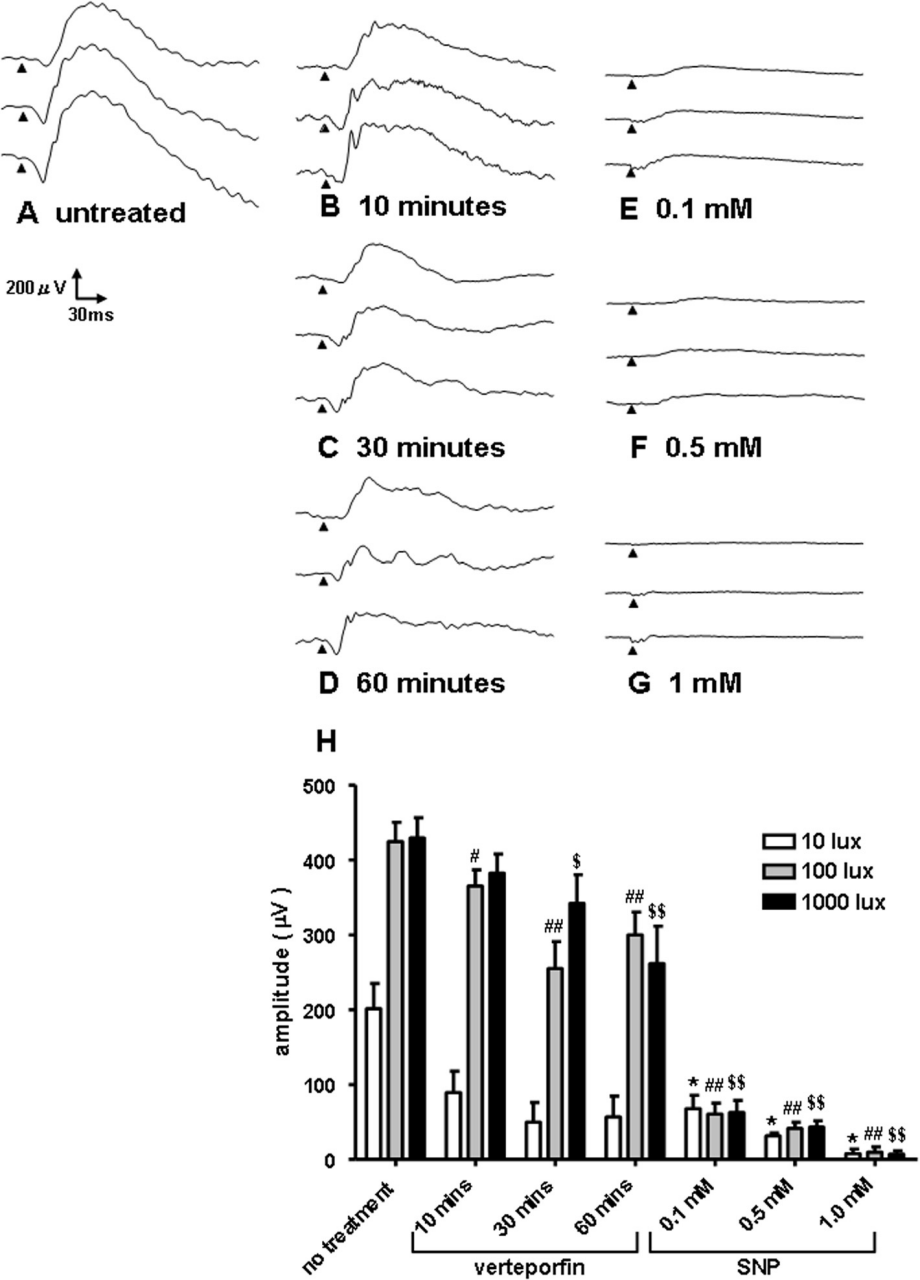
**The typical waveforms of electroretinogram (ERG) responses evoked by a white flash (duration: 10 ms; light intensity: 10, 100, and 1000 lux, top to bottom).** Waveforms with no treatment are shown in **A**. Eyes were submitted to PDT with light exposure (**B**: 10 min, **C**: 30 min, **D**: 60 min) or SNP injection (**E**: 0.1 mM, **F**: 0.5 mM, **G**: 1 mM). Comparison of b-wave amplitudes in eyes treated with either verteporfin or SNP (**H**). Data are shown as mean ± S.D., n = 3, *, #, $; p < 0.05, **, ##, $$; p < 0.01, unpaired *t*-test.

Amplitudes (a- and b-waves) of ERGs in verteporfin-treated eyes (Figure 2B-D) were decreased slightly compared to those in the untreated eye (Figure 2A). The b-wave amplitude declined with the duration of light exposure. In SNP-treated eyes, amplitude decreased with increasing SNP concentrations (Figure 2E-G). Even after injecting only a 0.1-mM solution of SNP (Figure 2E), ERG b-wave amplitudes were decreased markedly (Figure 2H).

**Figure 3:**
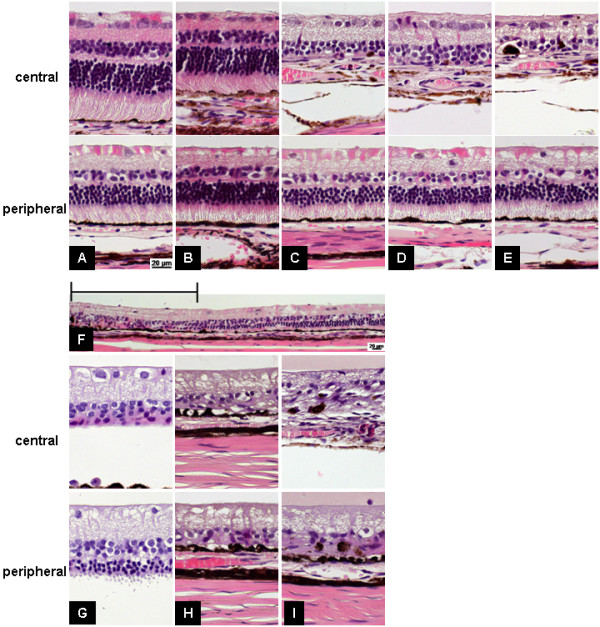
**Histological evaluations of photoreceptor degeneration after either PDT treatment or SNP injection.** Retinal histology without treatment is shown in (**A**). PDT was performed with 0.5 mg/kg verteporfin and a halogen reflector lamp for 0 (**B**), 10 (**C**), 30 (**D**), or 60 (**E**, **F**) min. The bar in the figure (**F**) indicates the area exposed to light. One hundred microlitters of SNP (**G**: 0.1 mM, **H**: 0.5 mM, **I**: 1 mM) are injected intravitreously injected into a rabbit eye.

Verteporfin treatment without light exposure did not induce photoreceptor degeneration (Figure 3B). However, degeneration of the neural retina (primarily photoreceptors) occurred after as little as 10 min of light exposure (Figure 3C–F). In the SNP-induced degeneration model, the lesion included the inner and photoreceptor layers of the peripheral retina as well (Figure 3G-I).

## Discussion

The photosensitising dye verteporfin is widely used as part of photodynamic therapy (PDT) for the treatment of CNV. However, PDT is not purely selective for the choroid and may induce damage to the retina [18]. In primates [20,21] and rabbits [22], PDT induces dose-dependent damage to photoreceptors and RPE cells. Following the absorption of specific wavelengths of energy [23], verteporfin generates oxygen radicals, which are toxic to photoreceptor and RPE cells. In our study, fundus photography revealed that the photoreceptor damage triggered by exposure to light after the intravenous injection of verteporfin was limited to areas that were exposed to light.

NO is released from SNP primarily through photochemical reactions [24] and by various reducing metabolites including thiols, which are contained in biological organelles such as microsomes [25]. NO is involved in numerous retinal functions [26,27]. Endogenous NO enhances the cone response during light-adaptation [28,29], sometimes leads to retinal toxicity (e.g., photoreceptor degeneration) [30] and contributes to ischemia-induced injury [31-33]. Under normal conditions, in contrast to ischemia or continuous or intense light exposure, the severity of induced retinal degeneration depends on local NO levels. The primary target of NO toxicity seems to be the outer retinal layers; even low-dose applications of NO (0.1 mM) induced degeneration in the outer retinal layers. The mechanisms of the degeneration induced by NO remain unclear. Several reports have outlined the relationship between NO and phagocytosis in RPE cells [34]. Becquet et al. [35] showed that NO inhibits the cGMP-independent phagocytosis of photoreceptor outer segments in vitro. Our laboratory found that NO inhibited cathepsin S activity via S-nitrosylation [27], which resulted in the accumulation of lipofuscin [36]. The degeneration induced by preventing rod outer segment phagocytosis is most likely gradual. Other factors may come into play to accelerate the process. Peroxynitrite formed from NO and the superoxide anion (O2-) or hydroxyl radical (OH-) has highly toxic effects on neuronal cells [37]. The excessive consumption of oxygen by photoreceptors generates high levels of superoxide anion (O_2_^-^) and hydroxyl radical (OH^-^). Free radicals accelerate photoreceptor degeneration; conversely, free radical scavengers inhibit the photoreceptor degeneration caused by continuous light exposure [38,39]. Peroxynitrite might be a major factor in the acute photoreceptor degeneration induced by NO. NO is a gas of low molecular weight; intravitreous injections are easily slowed by the viscous vitreous as they spread across the retina. However, once the gas has reached the retina, NO can pass through the inner retinal layers easily and cause toxic effects.

## Conclusions

Our results highlight 2 methods for the induction of photoreceptor degeneration in a rabbit model: verteporfin with light exposure and SNP. In the verteporfin-light exposure model, photoreceptor degeneration can be limited to the area exposed to light. This model is useful for the induction of local photoreceptor lesions, such as those that occur in age-related macular degeneration. In contrast, SNP induces photoreceptor degeneration throughout the entire retina. Both models are supported in large animals and may be useful for regenerative research, such as that directed toward the development of retinal prostheses, iPS transplantation, and gene therapy.

## Competing interests

The authors declare that they have no competing interests.

## Authors’ contributions

HI and ES obtained the fundus photographs and ERG recordings. NM prepared the histological sections. The data analysis was done by MT and HT. HT prepared the manuscript and designed the study. All authors read and approved the final manuscript.

## Pre-publication history

The pre-publication history for this paper can be accessed here:

http://www.biomedcentral.com/1471-2415/13/19/prepub
